# Acute Lymphoblastic Leukemia Transformation with a Precursor B-Cell Immunophenotype to Burkitt’s Lymphoma: a Case Report

**DOI:** 10.22088/cjim.14.4.760

**Published:** 2023

**Authors:** Masoumeh Jafari, Shahla Ansari Damavandi, Forugh Charmduzi

**Affiliations:** 1 Department of Pediatric Hematology and Oncology, Sschool of Medicine, Lorestan University of Medical Sciences, Khorramabad, Iran; 2 Department of Pediatrics, Ali Asghar Children’s Hospital, Faculty of Medicine, Iran University of Medical Sciences, Tehran, Iran; 3 Department of Pediatrics, Faculty of Medicine, Iran University of Medical Sciences, Tehran, Iran

**Keywords:** Precursor Cell Lymphoblastic Leukemia-Lymphoma, Burkitt Lymphoma, Immunophenotyping, Case report

## Abstract

**Background:**

As a new point, some very rare features can be revealed as initial diagnosis of acute lymphoblastic leukemia (ALL) without any evidence of lymphoma-like behavior which after initial recovery, presents with new evidence of lymphoma. Herein, a case of the immunophenotypes of blast cells in B-cell precursor acute lymphoblastic leukemia originated from MYC gene-related that was evidenced later by burkitt lymphoma feature.

**Case Presentation:**

Our case was initially diagnosed as a typical B-cell ALL cells with L1 morphology in peripheral blood smear and bone marrow aspiration that was not recovered and referred again that was finally featured as burkitt’s lymphoma with L3 morphological feature.

**Conclusion:**

Thus, in the primary diagnosis of B-cell ALL and especially in cases with treatment failure, the final feature of burkitt’s lymphoma should be potentially in mind.

The current standard diagnostic tool for the definitive diagnosis of acute lymphoblastic leukemia (ALL) integrated assessment of molecular and cellular morphology including genetics / cytogenetic , pathological behavior of cells and immune-phenotype according to the new classification released by the world health organization. These approaches are certainly employed to differentiate various types of leukemia from lymphoma. In this regard, the leukemic alternative indicates diffuse involvement of the peripheral blood and the bone marrow, while lymphoma is limited to nodal or extra nodal sites, with no or minimal involvement of the bone marrow (1).

 Even a rare type of leukemia named burkitt’s-type acute lymphoblastic leukemia (B-ALL), a variant of burkitt’s leukemia/lymphomathat originally taken from adults B-cell lymphoblast cells can be discovered (2, 3).

 Clinically, a combination of the clinical manifestations of lymphoma and leukemia such as fever, hepatosplenomegaly, pleural and abdominal effusion, as well as dramatically changes in blood cell counts can be observable in the affected patients. Thus, the differentiation of the pointed types of hematopoietic malignancies requires considering the multiple evidence approach including immunophenotyping, blood smear, and bone marrow examination and even chromosomal analysis (4). 

For instance, according to chromosomal analysis, B-ALL is mainly sourced from some chromosomal aberrations such as a transfer is included MYC gene as t (8;14)(q24;q32) leading functional over-expression of this gene (5). However, other gene aberrations involving chromosomes 1, 6, 7, 13, 17, and 22 can be discovered in relation to B-ALL (5). For instance, according to chromosomal analysis, B-ALL is mainly sourced from some chromosomal aberrations such as a transfer is included MYC gene as t(8;14)(q24;q32) leading functional over-expression of this gene (5). However, other gene aberrations involving chromosomes 1, 6, 7, 13, 17, and 22 can be discovered in relation to B-ALL (5). As a new point, some very rare features can be also revealed as initial diagnosis of ALL without any evidence of lymphoma-like behavior which after initial recovery, presents with new evidence of lymphoma. Herein, a case of the immunophenotypes of blast cells in B-cell precursor acute lymphoblastic leukemia originated from MYC generelated that was evidenced later by burkitt’s lymphoma feature. 

## Case Presentation

A 3.5-year old boy was admitted to our hospital and he complained of lassitude and fever. He was only child in family and they had no history of serious illness. There was no family history of cancer also his PMH was negative for any other disease. He displayed hepatosplenomegaly without lymphadenopathy.

 On admission, laboratory assessment showed dramatically raising lactate dehydrogenase (4315 u/l, normal range up to 480) as well as the following blood examination: red blood cell of 4.7 × 10^10^/L, serum hemoglobin concentration of 12g/dl, hematocrit level of 35.3%, platelet count of 129.000/mm^3^ and white blood cell count of 12.7×10^7^ with 48% of leukemic blasts in the cytoplasm. Leukemic blasts accounted for 50% of bone marrow cells which had mononuclear and typical B-cell ALL cells with L1 morphology was also revealed.

**Figure 1:**
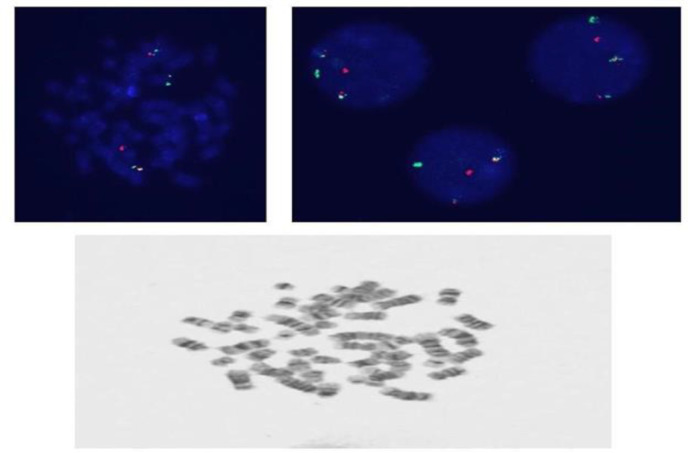
Clinical Data: FISH study for rearrangement of MYC/IGH, simultaneous Bone Marrow study BM17217-1

 An immunophenotype analysis revealed positivity for CD19, CD10, CD22, CD20, iCD79a, CD58, HLA DR and adversely negativity for terminal deoxynucleotidyl transferase and surface immunoglobulins, suggesting that the leukemic blasts had a precursor B-cell immunophenotype. A fluorescence in situ hybridization (FISH) (figure1) analysis revealed an positive for IGH/MYC fusion t(8;14)(q24;q32) in 42.8% corresponding to translocation between IGH(14q32) and MYC(8q24) gene regions. For therapeutic management, the patient was treated with induction therapy with prednisolone, vincristine, daunoromycin, L-asparginase along with consolidation therapy adjusted from a regime for standard risk leukemia based on to the pediatric leukemia study group BFM standard risk (SR-IR/ALL) with methotrexate and 6 mercapetopurin in 56 days. Then, re-induction therapy with dexamethasone, vincristine, doxorubicin, L-asparginase and cyclophosphamide was scheduled leading full recovery for eight months. However, the patient referred again after initial management and 8 months recovery period with fever and abdominal pain. In examination, right lower quadrant abdominal tenderness was found. Abdominal ultrasonography led to diagnosis of appendicitis with visible end loop of appendix with the diameter of 8mm and also an inflammatory mass in the right lower quadrant (phelegmon). In Abdominal CT scan: there was a calcified focus approximately measuring 6.5×5mm in RLQ associated with adjacent obvious fat stranding and non-significant mesenteric lymph nodes. These finding were suggestive of perforated appendicitis. 

Based on physical examination and imaging findings and following surgical consultation, the patient was treated with antibiotic therapy but with inappropriate therapeutic result and even worsening the symptoms. In next step, surgical exploration was planned showing a huge mass in right lower quadrant abdominal zone. 

**Figure 2:**
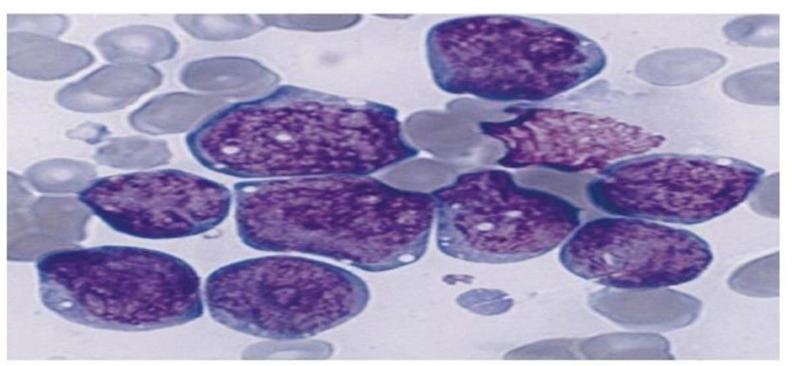
Diagnostic bone marrow aspiration photomicrographs for each c are characterized by large, regular blasts with deep blue vacuole cytoplasm, round to oval nuclei, fine to slightly coarse chromatin, distinct nuclei, and regular and fixed features in most malignant cells. Case # 1 differs from conventional L3 ALL in having somewhat larger chromatin, more prominent nuclei, and less regular cellular features

Then, bone marrow aspiration was performed that revealed L3 (figure2) morphology that was typical for burkitt lymphoma. A biopsy was also performed and the sample was sent for immunohistochemical chemistry (IHC) assessment that found a lymph proliferative neoplasm in favor with burkitt’s lymphoma. In addition, FISH hybridization was positive for Cmyc rearrangement. In final, by using treatment protocol for burkitt’s lymphoma (LMB 0281 protocol), recovery was achieved.

## Discussion

B-ALL with a precursor B-cell immunophenotype occurs in about 0.1% of all children with definitive diagnosis of ALL (1). In all affected patients, the detection of t(8;14) translocation is needed for recognition of B-ALL (5). The patients’ immunophenotypes of blast cells in B-cell precursor acute lymphoblastic leukemia are commonly planned for inductive therapy followed by consolidation chemotherapy. But, despite such therapeutic approach, the progression of B-ALL with a precursor B-cell immunophenotype seems to be worse than that of B-ALL with remained unknown reason. According to the literature, more than 60% of the patients recognized with B-ALL especially with additional chromosomal aberrations die or relapse (6, 7). In another case study, a 4-year-old man with a 2-month history of low back pain and weight loss was referred to the National Instituto de Caˆncer, Rio de Janeiro, Brazil in November 2004. After diagnosis as preBcell All and start chemotherapy he was in remission but in relapse shows burkit lymphoma histopathology. His clinical response showed that FAB L3 ALL with MYC rearrangement and an aberrant precursor B-cell phenotype is clinically same as BL ⁄ L. In addition, short, Intensive chemotherapy protocols is useful perhaps (8). In our case presented, q t(8,14) translocation was initially detected leading to definitive diagnosis of the immunophenotypes of blast cells in B-cell precursor acute lymphoblastic leukemia. Second, in spite of complete improvement of patients after induction followed by consolidation chemotherapy, our case faced with relapse short time after initial treatment with the evidences of severe abdominal inflammatory processes might be due to insufficient employed treatment protocol. As the first point, reviewing the literature indicates that by adding chimeric monoclonal antibodies such as rituximab, long-term proper therapeutic outcome is expectable and thus complete remission can be achieved (9). As another recommendation, an effective intervention would be stem-cell transplantation; however it potentially needs to HLA matching between donor and recipient (10). However, as revealed in our described case, tie final definitive diagnosis might be burkitt’s lymphoma that was initially mimicked by B-cell ALL. In other words, burkitt lymphoma is an insidious condition that may present with early-onset leukemia, which may lead to poor recovery from the diagnosis and inadequate follow-up treatment leading misdiagnosis, unfavorable treatment as well as significant complications.

To the best of our knowledge, the presented case is the first case of the transformation of ALL face to final lymphoma feature. However, some cases of B-ALL with a precursor B-cell immunophenotype and chromosomal variants have been previously described. We found one study described a similar case suffering B-ALL with a precursor B-cell immunophenotype with q t(8,14) translocation and also additional chromosomal aberrant as tetrasomy of 1q as well as t(2;4)(p13;q27) translocation. The patient was treated with dialysis before induction therapy followed by the consolidation therapy leading a proper 2-year remission (10). In another case described, a 45-year old male had fever, splenomegaly, sweating at night, and also prominent laboratory changes as raising uric acid and lactate dehydrogenase. LDH. Flow cytometry based on deficiency of cytoplasmic immunoglobulins, superficial immunoglobulins and light chain immunoglobulins showed neoplastic clone according to B-cell immunophenotype. Cytogenetic analysis revealed t(8;14)(q24; q32), the characteristic translocation of Burkitt's leukemia (11). Several similar cases of Burkitt's leukemia with B-cell immunophenotype precursors have been reported mainly in the children's literature. Summing the findings from the literature, it can be concluded that first BALL with a precursor B-cell immunophenotype may occur in all age subgroups. Second, the clinical manifestation of this phenomenon is nonspecific without diagnostic precision. Third, different chromosomal aberrations in addition to definitive displacement (t(8;14)) can be revealed that may be lead to poorer disease prognosis. Finally, although induction therapy followed by consolidation chemotherapy is the choice treatment approach for treating the affected patients; long-term remission may not be achieved unless adding monoclonal antibodies or stem-cell transplantation that can lead to long-term remission. In final and as a main point of our case presentation, burkitt lymphoma may be insidiously manifested by the B-cell ALL feature (L1 cellular morphology) that reveal its true faces in the long run and gradually. In the primary diagnosis of B-cell ALL and especially in cases with treatment failure, this feature should be potentially in mind.
